# Demand for mental health support services among health professionals in Vietnam: Lesson from COVID-19 Pandemic

**DOI:** 10.1371/journal.pone.0305869

**Published:** 2024-06-24

**Authors:** Nguyen Hoang Thanh, Than Manh Hung, Tran Nguyen Ngoc, Bui Van San, Doan Quoc Hung, Nguyen Dinh Hung, Vu Duc Binh, Le Hong Trung, Le Van Tinh, Nguyen Thanh Nam, Pham Huy Tan, Pham Tran Anh Khoa, Pham Ngoc Thach, Nguyen Tuan Khanh, Cao Duc Chinh, Nguyen Vu Trung, Tran Thi Mai Thi, Pham Ba Hien, Tran Anh Long, Nguyen Van Thuong, Nguyen Van Thanh, Tran Xuan Thang, Lai Duc Truong, Vu Quang Hieu, Satoko Otsu

**Affiliations:** 1 Hanoi Medical University, Hanoi, Vietnam; 2 School of Preventive Medicine and Public Health, Hanoi Medical University, Hanoi, Vietnam; 3 National Hospital of Tropical Diseases, Hanoi, Vietnam; 4 VNU University of Medicine and Pharmacy, Vietnam National University, Hanoi, Vietnam; 5 Department of Psychiatry, Hanoi Medical University, Hanoi, Vietnam; 6 National Institute of Mental Health, Bach Mai Hospital, Hanoi, Vietnam; 7 Department of Surgery, Hanoi Medical University, Hanoi, Vietnam; 8 Department of Cardiovascular and Thoracic Surgery, Viet Duc University Hospital, Hanoi, Vietnam; 9 Hanoi Medical University Hospital, Hanoi Medical University, Hanoi, Vietnam; 10 Hanoi Department of Health, Hanoi, Vietnam; 11 Clinical Hematology Department, National Institute of Hematology and Blood Transfusion, Hanoi, Vietnam; 12 Education and Healthcare Direction Center, National Institute of Hematology and Blood Transfusion, Hanoi, Vietnam; 13 Department of Health, Vinh Phuc Province, Vĩnh Yên, Vietnam; 14 Vinh Phuc Provincial General Hospital, Vinh Phuc Province, Vĩnh Yên, Vietnam; 15 School of Dentistry, Hanoi Medical University, Hanoi, Vietnam; 16 Ha Dong General Hospital, Hanoi, Vietnam; 17 Dong Da General Hospital, Hanoi, Vietnam; 18 Duc Giang General Hospital, Hanoi, Vietnam; 19 North Thang Long Hospital, Hanoi, Vietnam; 20 Disease Control and Health Emergency Program, World Health Organization Vietnam Country Office, Hanoi, Vietnam; Center for Research and Technology Transfer, VIET NAM

## Abstract

**Background:**

This study aimed to measure the preferences for mental health support among health professionals, their willingness to support the mental health of colleagues and associated factors.

**Method:**

A descriptive cross-sectional study was performed from August to October 2022 within five hospitals located in Hanoi, Vietnam. A total of 244 health professionals participated in the study. Data on socio-economic status, health and COVID-19-related characteristics, Depression Anxiety Stress Scale (DASS-21); and preferences for mental health support services were collected by using a structured self-reported questionnaire. Multivariate logistic regression models were utilized to identify associated factors with the demand for mental support services.

**Results:**

13.9%, 17.1% and 8.6% reported having at least mild depression, anxiety and stress, respectively. There 13.9% did not seek any mental health support during the COVID-19 pandemic. The most common support included talking with friends (52.9%), family (50.8%), colleagues (47.6%) and using social networks/Internet (43.5%). There 31.1% had been aware of mental health services, but only 18.0% used this service at least once. Regarding preferences, 47.3% had a demand for mental support services, and the most preferred service was providing coping skills (25.9%), followed by skills to support others against mental problems (22.2%). Major sources of support included psychiatrists (34.4%), colleagues (29.1%) and family (27.9%). The main preferred channels for support included telephone/mobile phone (35.7%) and Internet (20.9%). Only 12.3% were willing to provide mental support for colleagues during the pandemic. Age, education, perceived mental health status, ever seeking any mental service, and DASS-21 depression score were associated with demand for mental support services.

**Conclusion:**

This study found a lack of awareness of mental health services for health professionals, as well as moderate levels of demand for this service in this population. Raising awareness and developing tailored mental health support services are important to enhancing the mental well-being of health professionals in Vietnam to prepare for the next pandemic.

## Introduction

The impact of the COVID-19 pandemic on healthcare personnel has been widely acknowledged in the current literature. Numerous meta-analyses have consistently demonstrated an elevated prevalence of psychological disorders among these professionals [1,2]. There has been a notable increase in the prevalence of symptoms associated with mental disorders [3], and insomnia [4] after the onset of the COVID-19 pandemic.

The persistence of the pandemic and its accompanying intricate psychological strain raises concerns regarding the uncertain long-term ramifications of this predicament. On an individual level, enduring prolonged exposure to severe psychological stress can lead to enduring adverse effects; acute conditions that arise as a response to such stressors may become chronic, and psychological disorders may be accompanied by physiological comorbidities [5]. If these individuals are members of healthcare personnel, the personal consequences they experience can have significant detrimental impacts on national healthcare systems. A previous review found that during the COVID-19 pandemic, the quality of healthcare services in different European countries was significantly reduced [6]. The escalation of sickness absence rates and the burgeoning inclination of individuals to resign and explore alternative careers may ensue. This would potentially escalate the already prevailing concerns arising from personnel insufficiency and deteriorate the working environment, thereby engendering a detrimental cycle for the staff members who remain. The aforementioned factors, coupled with heightened fatigue and diminished capacity for endurance within the remaining personnel, would consequently lead to a decline in the standard of patient care [7].

Despite the availability of established and effective therapeutic interventions for a variety of mental disorders, healthcare professionals exhibit hesitancy in seeking assistance for their emotional distress, both before and during the pandemic [3,8]. Additionally, it is noteworthy that various forms of COVID-19-related stigma have a detrimental impact on the likelihood of individuals seeking necessary assistance [9]. Moreover, there is evident stigmatization associated with mental illness specifically within the medical field [10]. Furthermore, a prior review indicated that a variety of programs frequently provided support to health professionals’ mental well-being; nevertheless, they failed to acknowledge the importance of implementing strategies to guarantee the longevity and sustainability of the program [9]. Therefore, it is important to measure the preferences of mental health support among health professionals to develop tailored services that could be suitable for them not only in the COVID-19 pandemic but also in other pandemics in the long run.

Vietnam is among the countries that significantly suffered from the COVID-19 pandemic. Recent research showed that approximately half of healthcare professionals (49. 7%) exhibited signs of mild depressive symptoms, while 34. 0% demonstrated moderate symptoms of anxiety, and 49. 3% reported experiencing high levels of stress due to the COVID-19 pandemic [11]. However, to date, little evidence about the preferences for mental health support among health professionals in Vietnam. This study aimed to measure the preferences for mental health support among health professionals, their willingness to support the mental health of colleagues and associated factors.

## Materials and methods

### Study settings and participants

The present study employed a descriptive cross-sectional design from August to October 2022 within five hospitals located in Hanoi, Vietnam including the National Hospital for Tropical Diseases, Bac Thang Long Hospital, Me Linh Hospital, Duc Giang Hospital, Ha Dong Hospital and Dong Da Hospital. Participants included 1) healthcare professionals (e.g. physicians, nurses, technicians, midwifery) working in these hospitals, 2) directly or indirectly participated in treating COVID-19 patients, and 3) agreed to participate in the study and gave written/verbal informed consent. People who did not agree to take part in the study were excluded. We included all health professionals in these hospitals who were eligible for the study. A total of 244 health professionals participated in the study. The protocol of this study was approved by the Institutional Review Board of Hanoi Medical University (Code: CS2021_01/GCN-HDDDNCYSH-DHYHN)

### Data collection and measurement

A structured self-report questionnaire was employed to gather the data. The survey administered to the participants comprised four main sections, namely: (1) Awareness and preferences for mental health support services; (2) Socioeconomic status, (3) Health and COVID-19-related characteristics; (4) Mental health. Each questionnaire could be completed within 15–20 minutes. The questionnaire was first piloted with 10 health professionals and then revised based on their feedback regarding logical orders and text. The final version of the questionnaire was approved by the research team and the leader of hospitals.

### Outcome variables

***Awareness and preferences for mental health support services*:** In this study, participants were asked to answer six questions to measure their awareness and preferences for mental health support services among health professionals, as well as their willingness to support the mental health of colleagues. The questions were as follows: "Do you know any mental health services for health professionals during COVID-19?" aimed to assess participants’ awareness of available services. "Have you used any mental health support during the COVID-19 pandemic? Please specify," explored their personal utilization of such services. "Do you have any demand for mental support services during the pandemic?" and "Do you have any demand for sources of mental support services during the pandemic?" sought to understand their needs for mental health support. "Do you have any demand for channels of mental support service during the pandemic?" investigated their preferred methods for accessing support. Finally, "Are you willing to provide mental support for colleagues during the pandemic?" assessed their readiness to support their peers’ mental health.

### Predictor variables

***Socioeconomic characteristics*:** including age, gender, marital status, education, occupation and position in the hospital.

***Health and COVID-19-related characteristics*:** including severity of COVID-19 patients who respondents participated in treatment (none/mild/moderate/severe), perceived risk of COVID-19 infection (none/yes), having any morbidity (none/yes), perceived mental health conditions (very weak/weak/normal/good/excellent). The Fear of COVID-19 (FCV-19S) was used to evaluate the level of fear individuals might experience towards the COVID-19 virus [12]. The scale comprises seven items that assess emotional fear responses towards the ongoing pandemic. Participants were invited to provide their responses using a five-item Likert-type scale, wherein the scale ranges from 1 (indicating strong disagreement) to 5 (representing strong agreement). The cumulative score falls within the range of 7 and 35, where a greater aggregate score corresponds to a heightened level of fear towards COVID-19. The Cronbach’s alpha of FCV-19S was 0.768, suggesting acceptable internal consistency reliability [13]. We adapted this instrument from previous studies given that this instrument has been used widely in Vietnam [14,15].

***Mental health*:** The Depression Anxiety Stress Scale (DASS-21) is a psychometrically validated quantitative instrument designed to assess distress levels, encompassing depression, anxiety, and stress. This study employs a 4-point Likert scale to assess each item. The scale consists of ratings ranging from 0, indicating that the item did not apply to the participant at all, to 3, indicating that the item applied to the participant very frequently. The scores for Depression, Anxiety, and Stress are calculated by summing the scores of the corresponding sub-categories and then multiplying the total by a factor of 2. Depression stratification entails five distinct levels: normal (0–9), mild (10–13), moderate (14–20), severe (21–27), and very severe (28 and above). Anxiety is categorized into five levels, specifically normal (0–7), mild (8–9), moderate (10–14), severe (15–19), and very severe (20 and above). Stress can be categorized into five distinct levels, namely normal (ranging from 0 to 14), mild (from 15 to 18), moderate (from 19 to 25), severe (from 26 to 33), and very severe (34 and above). This instrument had been used among Vietnamese health professionals previously with good validity and reliability [16].

### Statistical analysis

Stata software version 16.0 was used for analyzing data. Descriptive statistics including frequency, percentage, mean and standard deviation were performed. Multivariate logistic regression models were utilized to identify associated factors with the demand for mental support services. A stepwise backward selection strategy was used to build regression models, with a p-value of log-likelihood <0.2. A p-value of less than 0.05 was used for statistical significance.

## Results

Of 244 health professionals, most of them worked in district hospitals (49.0%). The mean age was 35.2 (SD = 7.1) years old. The majority of them were female (68.6%), had a spouse (75.4%), had a university education or above (57.2%), were nurses/technicians/Midwifery (56.7%) and were staff (92.6%) (Table 1).

**Table 1:** Demographic characteristics of respondents.

	Freq. (n)	Percent (%)
**Hospital level**		
Central	86	35.1
District	120	49.0
Provincial	39	15.9
**Age group**		
23–29	54	23.0
30–39	125	53.2
> = 40	56	23.8
**Gender**		
Male	77	31.4
Female	168	68.6
**Marital status**		
Having spouse	184	75.4
Single	60	24.6
**Education**		
Below University education	104	42.8
University education and above	139	57.2
**Occupation**		
Physician	51	20.8
Nurse/technicians/Midwifery	139	56.7
Others	55	22.5
**Position**		
Manager	18	7.4
Staff	224	92.6
	**Mean**	**SD**
Age	35.2	7.1

Table 2 indicates that 31.1% had been aware of mental health services, but only 18.0% used this service at least once. Regarding preferences, 47.3% had a demand for mental support services, and the most preferred service was providing coping skills (25.9%), followed by skills to support others against mental problems (22.2%). Major sources of support included psychiatrists (34.4%), followed by colleagues (29.1%) and family (27.9%). The main preferred channels for support included telephone/mobile phone (35.7%) and Internet (20.9%). There was 20.1% preferring face-to-face counselling. Only 12.3% were willing to provide mental support for colleagues during the pandemic.

**Table 2:** Awareness and preferences for mental health support among health professionals.

	Freq. (n)	Percent (%)
**Knowing any mental health service**		
Don’t know	168	68.9
Know but do not use	32	13.1
Use at least once	44	18.0
**Demand for mental support services during the pandemic**		
None	128	52.7
Providing knowledge about mental health	52	21.4
Providing coping skills for any mental health problems	63	25.9
Providing skills to support others against mental health problems	54	22.2
**Demand for the source of mental support services during the pandemic**		
None	91	37.3
Psychiatrist	84	34.4
Family	68	27.9
Friends	54	22.1
Colleagues	71	29.1
**Demand for channels of mental support service during pandemic**		
None	96	39.3
Internet	51	20.9
Telephone/Mobile phone	87	35.7
Phone messages	39	16.0
Face-to-face	49	20.1
Other	3	1.2
**Willingness to provide mental support for colleagues during pandemic**		
No	193	87.7
Yes	27	12.3

Table 3 shows that 28.6% of health professionals directly participated in treating COVID-19 patients. There 51.4% perceived having a risk of COVID-19 infection, and the mean score of FCV-19S was 20.0/35 (SD = 5.1). Regarding health status, 18.2% reported having any chronic morbidities and 25.1% had a normal mental health condition or weaker. According to the DASS-21 scale, 13.9%, 17.1% and 8.6% reported having at least mild depression, anxiety and stress, respectively.

**Table 3:** Health and COVID-19-related characteristics of participants.

	Freq. (n)	Percent (%)
**The severity of COVID-19 patients who respondents participated in treatment**		
None (or indirect participation)	175	71.4
Mild	13	5.3
Moderate	27	11.0
Severe	30	12.2
**Risk of COVID-19 infection**		
None	119	48.6
Yes	126	51.4
**Having any chronic morbidity**		
None	198	81.8
Yes	44	18.2
**Perceived mental health**		
Normal or below	61	25.1
Good	144	59.3
Excellent	38	15.6
**Depression (DASS-21)**		
No	211	86.1
Yes	34	13.9
**Anxiety (DASS-21)**		
No	203	82.9
Yes	42	17.1
**Stress (DASS-21)**		
No	224	91.4
Yes	21	8.6
	**Mean**	**SD**
DASS-21 Depression score (0–21)	1.67	2.27
DASS-21 Anxiety score (0–21)	1.90	2.11
DASS-21 Stress score(0–21)	3.11	3.03
Fear of COVID score (5–35)	20.0	5.1

**Fig 1:**
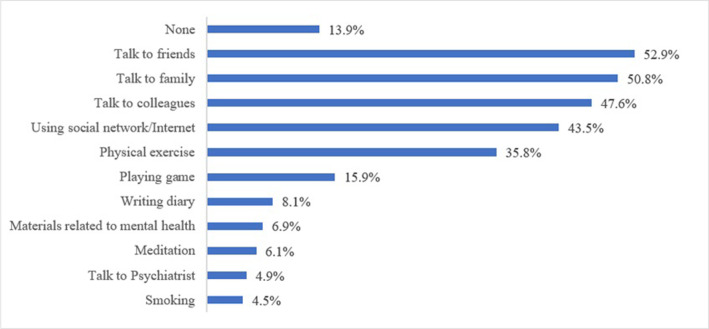
Mental health support among health professionals during COVID-19 pandemic.

Fig 1 illustrates that 13.9% did not seek any mental health support during the COVID-19 pandemic. The most common support included talking with friends (52.9%), family (50.8%), colleagues (47.6%) and using social networks/Internet (43.5%).

Table 4 indicated that higher age was associated with a lower likelihood of being aware of any mental service (OR = 0.95, 95%CI = 0.89–0.99). Meanwhile, having a university education or above (OR = 2.63, 95%CI = 1.33–5.22) and participating in treating severe COVID-19 patients (OR = 4.98, 95%CI = 1.06–23.38) were related to a higher odd of being aware of any mental service.

**Table 4:** Associated factors with preferences for mental health support among health professionals during the pandemic.

Characteristics	Being aware of any mental service		Having demand for mental support service		Willingness to provide mental support	
	OR	95%CI	OR	95%CI	OR	95%CI
Hospital level (Central vs Provincial)					2.43*	0.88; 6.72
**Age (years)**	0.95**	0.89; 0.99				
**Gender (Male vs. Female)**					0.45*	0.18; 1.12
**Education (< University vs. ≥ University)**	2.63***	1.33; 5.22				
**Occupation (Physicians vs. Nurses/technicians/Midwifery**	1.74*	1.00; 3.03	1.45	0.92; 2.29		
**Position (Manager vs. Staff)**	3.63*	0.94; 14.01				
**The severity of COVID-19 patients who respondents participated in treatment (vs. None)**						
Mild	1.39	0.38; 5.13				
Moderate	0.61	0.22; 1.67				
Severe	4.98**	1.06; 23.38				
**Perceived mental health (vs. Normal or below)**						
Good	0.43*	0.18; 1.01	0.97	0.47; 2.01		
Excellent	0.89	0.27; 2.91	4.42**	1.39; 14.11		
**Seeking any mental health support (None vs. Yes)**			5.26***	1.74; 15.93		
**Fear of COVID-19 scale score**	0.95	0.89; 1.02				
**DASS-21 Depression score**			0.74***	0.63; 0.87	1.14**	1.01; 1.30
**DASS-21 Stress score**	1.12*	0.98; 1.28				

*** *p<0.01*

** *p<0.05*

* *p<0.1*.

Health professionals perceiving excellent mental health (OR = 4.42, 95%CI = 1.39–14.11) and seeking any mental support during the pandemic (OR = 5.26; 95%CI = 1.74; 15.93) were more likely to have a demand for mental support services. Meanwhile, a higher DASS-21 depression score was negatively associated with having a demand for mental support services (OR = 0.74, 95%CI = 0.63; 0.87). Similarly, a higher DASS-21 depression score was negatively related to being willing to provide mental support for colleagues (OR = 1.14, 95%CI = 1.01; 1.30).

## Discussion

Our study contributed to the knowledge regarding preferences for mental health care services among health professionals during the pandemic, which could be a lesson for other pandemics in the future. This study showed a lack of awareness of mental health services for health professionals, as well as moderate levels of demand for this service in this population.

The global outbreak of the COVID-19 pandemic has been associated with considerable psychological ramifications among healthcare professionals [2]. Frontline healthcare professionals have encountered the adverse health conditions of individuals afflicted with COVID-19, alongside the unfortunate demise of patients [17]. In this study, the rates of at least mild depression, anxiety and stress were 13.9%, 17.1% and 8.6%, respectively, which were lower than other reports. For example, a recent meta-analysis found that approximately 33% of healthcare workers reported depressive symptoms, 42% exhibited anxiety features; approximately 40% experienced acute stress and 32% exhibited post-traumatic symptoms [2]. Our results were also lower than a prior online survey which showed that 49.7% of healthcare professionals (49.7%) had mild depressive symptoms [11]. However, findings in this study aligned with a previous study in two healthcare facilities, which indicated that the prevalence of stress, anxiety and depression in the first wave of COVID-19 was 8.0%, 17.5% and 14.8%, respectively [16]. The relatively low prevalence of mental health issues among Vietnamese healthcare workers, compared to their counterparts in other nations, may be linked to Vietnam’s comparatively lower COVID-19 infection and mortality rates, as reported by the World Health Organization. Furthermore, the data collection period (August to October 2022) likely didn’t overlap with the pandemic’s peak, leading to substantially less stress on healthcare professionals than during the most critical periods of the outbreak. However, this also suggests that the mental health status of health professionals in health facilities has not improved, despite our study being conducted when the COVID-19 pandemic was under control.

This study found that 13.9% of participants didn’t seek any mental health support during the COVID-19 pandemic, and about 60% weren’t aware of mental health services. Our observations also revealed that individuals facing significant mental strain didn’t express a need for external support, possibly because they felt they had enough on their own. Moreover, there seems to be a prevailing attitude within the healthcare community that discourages people from seeking help and openly discussing mental health issues, making it harder for those in urgent need to get assistance. The hesitation to seek mental health care may come from a busy schedule, limited knowledge or recognition of mental health issues, or a lack of accessible resources. One effective solution involves using the internet, social media, and other online platforms, paired with word-of-mouth recommendations. This approach has replaced traditional therapy sessions with psychologists and doctors due to its easy access, convenience, and lack of COVID-19 exposure. Given the hectic and unpredictable schedules of COVID-19 frontline workers in Vietnam, mental health support should include online resources like recorded therapy sessions or mindfulness blogs available anytime. Mental health experts and organizations use these platforms to share educational information, provide support, and promote mental health services.

The recommendations drawn from the findings emphasize three key areas to effectively support medical staff amid the current challenges while also preparing Vietnam’s healthcare system for future crises. On the individual level, providing mental health support through resident psychiatrists, support groups, and mindfulness sessions can address psychological distress and improve staff retention. Cognitive-based therapy, both individual and group, has been shown to reduce anxiety and depression in healthcare professionals facing crises. Organizationally, ensuring PPE availability and standardized training can bolster healthcare workers’ resilience. Mentorship programs and improved staff allocation, especially in intensive care units, can promote workplace solidarity while providing sufficient breaks. At the national level, governments should partner with local media to reduce stigma and share accurate health information. Engaging the public to disseminate health messages effectively is vital, while offering grief counseling for families and psychological support for patients can alleviate the pressure on frontline workers. These measures, paired with seeking healthcare professionals’ input, will help create a comprehensive and proactive framework to address future crises.

In the present study, various limitations were encountered and need to be acknowledged. Initially, the research was undertaken after the emergence of the second wave of the COVID-19 outbreak in Vietnam, during a time when the prevalence and fatality rates associated with COVID-19 were minimal. The aforementioned circumstance potentially contributed to the decrease in prevalence rates of mental health disorders observed among healthcare professionals in our study. Another limitation of this study was the small sample size due to resource constraints in conducting the research. Additionally, since the study involved doctors treating COVID-19 patients, scheduling the participation of healthcare professionals was also a significant challenge. Moreover, it is important to note that although this was a multi-centre study, the healthcare workers involved in the research were selected exclusively from Hanoi, thereby potentially failing to accurately depict the broader spectrum of healthcare personnel in Vietnam. Additional research should be conducted using a larger sample size and a more extensive selection of hospitals across all regions in Vietnam. Finally, a notable gap exists between the proportion of participants who were frontline healthcare workers (29.6%) and those who would be expected to have higher rates of mental health issues due to contact with COVID-19 patients. The low percentage of frontline workers might have impacted the overall reported prevalence of depression, anxiety, stress, and help-seeking behaviors in the study.

## Conclusion

This study found a lack of awareness of mental health services for health professionals, as well as moderate levels of demand for this service in this population. Raising awareness and developing tailored mental health support services are important to enhancing the mental well-being of health professionals in Vietnam to prepare for the next pandemic.
